# The Effect of mHealth on Exclusive Breastfeeding and Its Associated Factors Among Women in South Ethiopia: A Cluster Randomized Controlled Trial

**DOI:** 10.3390/nu17213477

**Published:** 2025-11-05

**Authors:** Girma Gilano, Andre Dekker, Rianne Fijten

**Affiliations:** 1 Department of Public Health Informatics, School of Public Health, College of Medicine and Health Sciences, Arba Minch University, Arba Minch P.O. Box 21, Ethiopia; 2 Department of Radiation Oncology (Maastro), GROW Institute for Oncology and Reproduction, Maastricht University Medical Centre+, 6229 ER Maastricht, The Netherlands; andre.dekker@maastro.nl (A.D.); rianne.fijten@maastro.nl (R.F.)

**Keywords:** EBF, mHealth, child feeding, SMS

## Abstract

Introduction: Exclusive breastfeeding (EBF) is vital for optimal infant health, reducing the risk of infections and enhancing cognitive development. Despite WHO’s recommendation of EBF for the first six months of life, global adherence remains suboptimal, particularly in low-resource settings. This study evaluates the impact of mobile health (mHealth) interventions on exclusive breastfeeding (EBF) among mothers in South Ethiopia. Methods: A cluster randomized controlled trial was conducted in the Gamo Gofa zones, South Ethiopia, involving 20 health facilities (10 intervention and 10 control). The study included 680 pregnant mothers recruited using simple random sampling from antenatal care (ANC) registers and family folders. Mothers in the intervention group received mHealth support, including breastfeeding information and reminders, while the control group received standard care. Participants were followed from the second trimester to six months postpartum. Multilevel survival analysis was applied to assess EBF duration, and multilevel logistic regression was used to evaluate complementary feeding within the first month. Results: The intervention group had a significantly higher probability of maintaining EBF at six months than the control group (AHR = 0.40, 95% CI: 0.26–0.62, *p* < 0.001). The secondary outcome also shows higher odds of early breastfeeding initiation in the intervention group (AOR = 4.71, 95% CI: 3.10–7.16, *p* < 0.001). ANC frequency was associated with a lower hazard of stopping EBF (AHR = 0.87, 95% CI: 0.79–0.97, *p* <0.05). The presence of diarrhea was associated with a high hazard of EBF (AHR = 1.47, 95% CI: 1.07–2.02, *p* < 0.05). College and above partner education was associated with high hazards of EBF (AHR = 2.41, 95% CI: 1.01–5.78, *p* < 0.05). The random effects variance (Var = 0.10, 95% CI: 0.01–0.07) indicated significantly lowered cluster-level variability. Conclusion and Recommendations: The mHealth intervention significantly improved EBF adherence and early breastfeeding initiation among mothers in South Ethiopia. Early breastfeeding, ANC frequency, and family size were protective factors, while high partner education and diarrhea disease increased the risk of early cessation of EBF. These findings highlight the potential of mHealth in addressing key barriers to EBF. Scaling up similar interventions, focusing on high-risk groups, could enhance adherence to WHO’s breastfeeding recommendations and improve maternal and child health outcomes in resource-limited settings.

## 1. Introduction

Exclusive breastfeeding (EBF), defined as feeding an infant only breast milk without additional liquids or solids except for necessary medications or vitamins, is a cornerstone of optimal infant nutrition and health [1,2]. The World Health Organization (WHO) recommends EBF for the first six months of life due to its significant benefits, including improved infant immunity, reduced risk of infections, and enhanced cognitive development. Despite these advantages, global rates of EBF remain suboptimal, with many countries falling far below the WHO target of 50% EBF by 2025 [2,3]. In Ethiopia, recent data indicate that EBF rates are approximately 60%, but adherence to the six-month recommendation is often compromised, especially in rural areas [4].

Mobile health (mHealth) interventions have emerged as innovative strategies to address maternal and child health challenges, such as promoting EBF [5,6,7,8]. By leveraging mobile technology, mHealth can provide mothers with real-time health information, reminders, and support [9], which are especially beneficial in low-resource settings where access to healthcare facilities is limited [10]. Evidence from various randomized controlled trials highlights the potential of mHealth interventions to improve breastfeeding practices by addressing common barriers such as lack of knowledge, cultural misconceptions, and inadequate support [11,12].

In Ethiopia, the widespread use of mobile phones offers an opportunity to implement mHealth solutions to improve maternal and child health outcomes [6,7]. Despite high mobile subscriptions, access to a stable mobile network remains limited in some rural areas, presenting challenges for implementation [13]. As of 2024, Ethiopia is East Africa’s largest telecom market, with about 87 million mobile subscriptions—roughly 28% of the region’s total [13]. This suggests that mobile-based service delivery could reach a substantial portion of the population. Evidence on the effectiveness of mHealth interventions for promoting exclusive breastfeeding (EBF) in Ethiopia is limited. However, studies from similar low- and middle-income countries indicate that tailored mHealth interventions can significantly improve breastfeeding duration and adherence to WHO recommendations [12,14,15].

This cluster-randomized controlled trial aimed to evaluate the effect of mHealth on EBF among mothers in the Gamo Gofa zone, South Ethiopia. Specifically, the study sought to determine whether providing health information and reminders through mobile phones improves EBF rates at six months postpartum compared to standard care. Additionally, the study investigated factors associated with EBF, including maternal education, breastfeeding initiation, and family size. By addressing these objectives, the study contributes to the growing body of evidence on mHealth interventions and their role in enhancing maternal and child health in resource-limited settings.

## 2. Methods and Materials

### 2.1. Study Design and Setting

This study was a cluster-randomized controlled trial conducted in the Gamo Gofa zones, South Ethiopia. The Gamo and Gofa zones share similar cultures and lifestyles, having lived together for centuries as a single ethnic community. The two zones are conveniently selected for their proximity. A cluster-randomized trial was conducted across 20 health facilities, with 10 facilities allocated to the intervention arm and 10 to the control arm. The primary participants were pregnant women attending antenatal care (ANC) services at these health facilities and in surrounding communities. Participants were followed from the end of the second trimester through six months postpartum to evaluate the impact of mobile health (mHealth) interventions on exclusive breastfeeding (EBF). This study commenced on 1 May 2024, with data collection for this objective beginning on 31 January 2025. The study includes multiple outcomes, including postpartum family planning, exclusive breastfeeding, prelacteal feeding, and childhood vaccinations. We have separated these outcomes into distinct article reports. The outcomes pertain to services provided during the antenatal care (ANC) and postnatal care (PNC) periods and may not exhibit direct correlations with one another. 

### 2.2. Study Participants

#### 2.2.1. Eligibility and Exclusion Criteria

Initially, we randomly selected maternal and child health facilities from the available 102 institutions. Within these facilities, we recruited pregnant women who met the following criteria: age 18 or older, residing in the nearby community, literate (able to read/write), had used a mobile phone, and gestational age between 16 and 28 weeks at recruitment. Exclusion criteria included: plans not to breastfeed, high-risk pregnancies requiring special care, non-residents likely to move away before the study’s completion, or likely to experience network interruptions.

Participants were required to remain in the study from recruitment (during pregnancy) through six months postpartum to assess outcomes. If a participant moved away and could not be reached for intervention or data collection, she was considered lost to follow-up.

The gestational age range at recruitment was 16 to 28 weeks, and recruitment lasted two months. At the start of the intervention, gestational age was between 24 and 28 weeks. The maximum gestational age at the beginning of the intervention was 28 weeks. All mothers meeting these eligibility criteria were included. The study selected literate mothers for the mHealth intervention to ensure clear comprehension of SMS content and to attribute outcome differences solely to the intervention. Relying on others to read personal health information could compromise privacy and introduce variability in message delivery.

Context note: Ethiopia has the largest telecom market in East Africa, with 70% mobile subscriptions among the total population, as reported in Revenue Magazine’s Africa Telecom 50 Report 2024 [16]. Although restricting participation to literate mothers may be a limitation, this approach could pave the way for future studies to modify and use audio or video delivery to include illiterate participants.

#### 2.2.2. Sampling and Recruitment Process

Health facilities were initially randomized into either an intervention or a control group. Within the selected facilities, participants were recruited through simple random sampling from antenatal care (ANC) registers and family folders, and then eligible mothers were enrolled. The required sample size was calculated to be 680 mothers, based on findings from a previous study in which vaccination coverage was 70.9% among the control and 82.6% among the intervention group [17]. The calculation assumed an intraclass correlation coefficient (ICC) of 0.011 [18], a minimum detectable difference in prelacteal feeding prevalence of 12%, 80% statistical power, a 95% confidence interval, and a 5% significance level (alpha). Each cluster (health facility) contributed approximately 34 to 35 mothers (mostly 35), ensuring sufficient power to identify meaningful differences between groups.

$$m=\frac{{\left(Z_{1-\alpha /2}\;+Z_{1-\beta }\right)}^{2}\left[P_{1}\left(1-P_{1}\right)+P_{2}\left(1-P_{2}\right)\right]}{\Delta^{2}}\times [1+(n-1)\rho ]$$

where [1 + (n − 1) ρ] is the design effect multiplied by ICC (ρ), n is the number of women per cluster

∆ = P_1_ − P_2_

Zx = is the x’s percentage of the Xth percentage point of the standard normal distribution, Δ is the clinically important difference in treatment means.

- P_1_ (proportion of fully vaccinated children among controls = 70.9%
- P_2_ (proportion of fully vaccinated children among intervention groups = 82.6%
- Delta, the difference in the proportion of fully vaccinated among groups = 12%
- α (level of significance) = 5%
- The standard normal curve or Z at 95% CI and 5% of α= 1.96 (two-tailed)
- Power of study = 80%
- Control to intervention ratio = 1:1

A total of 672 participants were required to achieve 80% power to detect a 12 percentage-point difference in fully vaccinated children between groups at α = 0.05 (two-sided). During recruitment, 680 participants were enrolled to ensure adequate power in the face of potential loss to follow-up and data quality issues; all randomized participants were included in the primary analysis.

### 2.3. Randomization and Blinding

Participant selection involved two-stage randomization. In the first stage, health facilities were randomized into intervention and control groups using sequentially numbered pieces of paper that contained the names of HFs placed in an opaque envelope. The study included two primary hospitals, one serving as a control and the other as the intervention site. Health posts were not selected individually; instead, when a health center was randomly chosen for inclusion, all its associated health posts were automatically incorporated into the study. Randomizations were conducted at the facility level to minimize contamination between groups.

**Figure 1:**
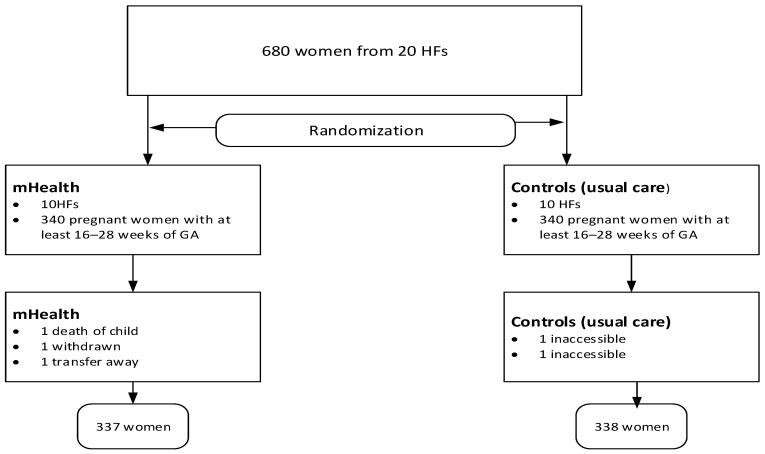
Study flow chart showing study procedures.

In the second stage, mothers were selected randomly from family numbers in family folders and ANC registers. Due to the nature of the mHealth support, mothers were not blinded to the intervention, but data collectors and analysts were blinded to group assignment to minimize bias. Mothers were also unaware of the research objective following the SMS intervention. The detailed randomization process is also described elsewhere in the protocol [19]. Although the protocol provides detailed information, some modifications have been made since its initial publication before commencement (Figure 1).

### 2.4. Intervention

Mothers in the intervention group received tailored health information messages and reminders via their mobile phones. We established the implementation server at the Arba Minch University in South Ethiopia. Twenty health facilities that provide maternal and child services are included. Women in intervention receive messages for months. The ANC and PNC attendance were the strategic points for gaining and following women. The trial aims to increase breastfeeding, PPFP, enhance timely childhood vaccination and nutrition awareness, and reduce prelacteal feeding. The intervention was targeted to empower women through timely, action-oriented health information. Pregnant women aged ≥18, gestational age 16 weeks at the beginning of recruitment, literate, able to use a mobile phone, and residing in the catchment area, enrolled from health centers or primary hospital, health posts (via ANC registers and family folders). The women started receiving messages at the gestational age of 24 to 28 weeks. The message focuses on ANC, PPFP, vaccination, breastfeeding/nutrition, tips for maintaining exclusive breastfeeding, danger signs, partner/community engagement, and reminders. We developed the intervention based on the WHO guidelines [20], UNICEF breastfeeding guideline [21], national Reproductive, Maternal, Newborn, Child Health, and Family Planning (RMNCH/FP) strategies, and behavioral change communication frameworks (BCCI) [22]. For example: “Exclusive breastfeeding builds your baby’s immunity. No other foods or liquids needed.” This translates to Amharic as “ጡትን ብቻ ማጥባት የበሽታ መከላከል አቅምንን ይገንባል። ከጡት ውጪ ሌላ ምንም ምግብ አያስፈልግም”. Since all participating women can read and write Amharic, we translated all messages into Amharic. Each message is reviewed for its cultural appropriateness and the health literacy level of participating women. The FrontlineSMS application was customized and monitored by a computer scientist team member who acted as a trial manager. The app was set to deliver messages at 7:00 AM local time. The trial manager also monitored the message delivery and reading status (whether the mother read or opened the message). The system automatically schedules or queues messages for retry if the first attempt fails, within a time range from 7:00 AM to 11:00 PM local time. If the mother is no longer in the message receivers and is inaccessible on a voice call, she is registered as lost to follow-up. Health information messages were sent biweekly (every two weeks) for 9 months without interruption. Reminder messages were sent one day before each appointment. For this study, an automated one-way message was sent to participants.

### 2.5. Control

The control group received standard antenatal, delivery, postnatal, and vaccination services unchanged as usual care. The SMS reminder intervention served as supplementary support, not a replacement for essential care. The trial was justified by scientific uncertainty regarding the efficacy of SMS-based mHealth reminders for enhancing vaccination uptake in these contexts, with both groups receiving identical healthcare and health education.

The trial aims to increase maternal service utilization, postpartum family planning (PPFP) uptake, timely childhood vaccination, and exclusive breastfeeding rates through tailored mobile phone–based messaging. Mothers in the control group received standard care as usual, which included routine health education provided during ANC visits without additional mHealth support. For postpartum outcomes, we sent a total of 6760 messages with a fidelity rate of 98.5%. Loss to follow-up was 0.73%. Additionally, mothers who did not read the messages reduced the overall fidelity of the intervention. A trial manager was responsible for uploading messages created by the authors from prior studies. The study employed a secure VPN, but centralized systems managed by a government agency, such as a national or regional SMS gateway, were recommended for automatic message sending to reduce the workload of local staff. Simpler mobile-based dashboards are a cost-effective alternative to VPNs for supporting mHealth in rural health extension programs. In Ethiopia, internet access and health facility-level technicians can help address skill gaps.

## 3. Study Variables

### 3.1. Outcome Variables

Primary Outcome: Exclusive breastfeeding (EBF) status at six months postpartum. The study analyzed the event variable of EBF in the sixth month and the time to stop EBF as a time variable.

Failure = 1: The event of interest occurs (stopping EBF before the 6th month).

Failure = 0: The event does not occur (continuing EBF for 6 months or being censored).

Censor = A situation where the event of interest (stopping EBF failure from occurring) has not happened for some study subjects by the end of the study period or the time of analysis

Secondary Outcome: Prevalence of complementary feeding within the first month after birth.

We coded mothers who started complementary feeding in the first month as ‘Yes’ (1) and those who did not as ‘No’ (0).

Independent Variables: Residence (rural and urban), Diarrhea (yes/no), Time to First PNC (number), Provider (Nurse, Midwife, Health officer, Doctor, Health extension worker), BF in first 1 hour (yes/no), Age of an infant (number), Colostrum only for 3 days (yes/no), Bottle-feeding, (yes/no), Complimentary feeding at first month (yes/no), Complementary feeding at fifth month (yes/no), Maternal education (read/write, 1–8 grade, 9–12 grade, college/above), Monthly income (number), Partner education (read/write, primary, secondary, college/above), Partner occupation (farmer, gov’t employ, merchant, self-employ, other), mode of delivery, Pregnancy plan (yes/no), Partner support (yes/no), Nutritional counseling (yes/no), Distance to nearest HF (number), Religion (Ethiopian Orthodox Church, Protestant, Muslims), Maternal occupation (farmer, housewife, gov’t employ, merchant, self-employ), Information source (Mass media (TV, Radio), Healthcare providers), ANC (number), Number of children (number), and Family Size (number). Since these are the combined variables of the whole study, we present only the related variables with a specific outcome.

#### Data Collection and Follow-Up

Data collection occurred at three time points: during pregnancy (baseline information), at the end of the first month (prelacteal feeding), and at six months postpartum (exclusive breastfeeding, postpartum family planning, and child vaccinations). The EBF data were collected alongside postpartum family planning and childhood vaccinations. Participants were followed from enrolment during pregnancy until the infant’s sixth month.

Trained data collectors used structured questionnaires to gather information on breastfeeding practices, maternal and child health, and socio-demographic characteristics. To ensure accuracy, all data were double-entered into a secure database. The collection was both phone-based and face-to-face interviews. We pre-tested the questionnaire on health facilities outside the study area. The data collection tool was derived from previous studies on mHealth in Africa and Ethiopia [19,23,24,25].

### 3.2. Statistical Analysis

All analyses were performed in STATA version 14, with significance set at a *p*-value of <0.05. The primary analysis for EBF was conducted using multilevel survival analysis to account for time-to-event data and clustering at the health facility level. The analysis excluded mothers who were either lost to follow-up or inaccessible at the start of the intervention. The study applied multilevel logistic regression to analyze the secondary outcome of complementary feeding in the first month. Model performance was evaluated using the intraclass correlation coefficient (ICC), Akaike Information Criterion (AIC), Bayesian Information Criterion (BIC), Log-likelihood, model variances, and deviance. Weibull demonstrated superior model performance compared to other models such as Exponential, Gompertz, Loglogistic, Lognormal, and Generalized Gamma. The multilevel logistic regression consisted of a sequence of models: (1) the null model (intercept-only), (2) the random-effects model (group-level analysis), (3) the fixed-effects model (individual-level analysis), and (4) the mixed-effects model (a combination of group- and individual-level predictors). The null model does not include the predictors. The random effect model incorporates group-aggregated variables, such as community women’s education, occupation, and residence. The fixed-effects model includes individual-level characteristics, such as sociodemographic and socioeconomic variables. The mixed-effects model combines random and fixed effects. Since data for exclusive breastfeeding (EBF) were collected alongside childhood vaccinations and postpartum family planning, we extracted EBF-related variables and analyzed them separately.

## 4. Results

Overall, 675 mothers with newborns completed follow-up, yielding a dropout/lost-to-follow-up rate of 1%. On average, infants were six months old at the time of this collection. Five percent of mothers had cesarean deliveries. The intervention groups achieved 73% exclusive breastfeeding (EBF), and the diarrheal disease incidence was 15%. In the control groups, 51% practiced EBF, 21% experienced diarrheal disease, and 33% used bottle feeding (Table 1).

### 4.1. Survival Probabilities over Time

**Figure 2:**
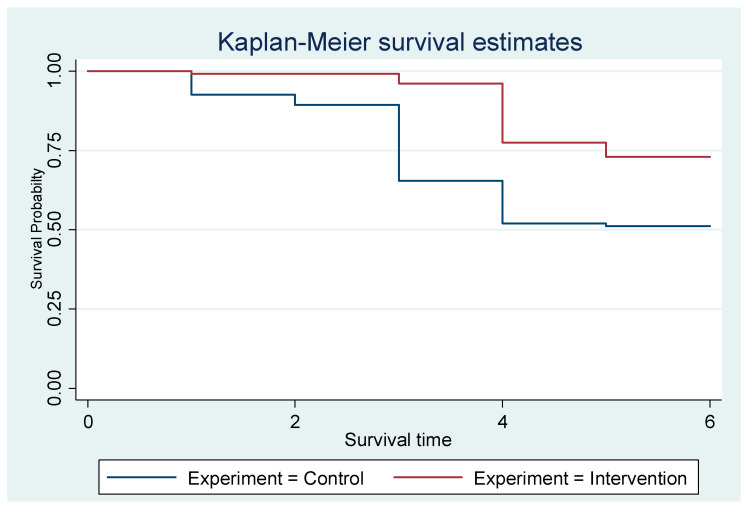
The Kaplan–Meier showing the exclusive breastfeeding estimates of the groups.

Both interventions and controls start at a survival probability of 1.0 (100%), as all mothers are exclusively breastfeeding at the beginning. However, the intervention group maintained a higher survival probability than the control group throughout the six months. The control group shows a steeper decline in survival probability, indicating that mothers in this group stop EBF rapidly. By the end of the observation period (six months), the survival probability for the intervention group is noticeably higher than for the control group (Figure 2).

#### 4.1.1. Survival Curves for Time to EBF Cessation by Group

**Figure 3:**
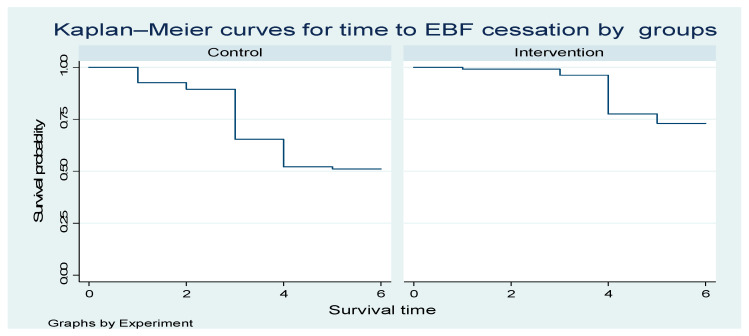
The Kaplan—Meier curves for time to EBF cessation intervention and controls.

Figure 3 below shows one curve for the Control group and one for the Intervention group. The y-axis represents the probability of remaining in exclusive breastfeeding (i.e., not yet ceased EBF) over time. The x-axis represents time in months since birth. The higher the curve, the higher the probability of still exclusively breastfeeding at a given time point. The intervention curve is consistently above the Control curve; the intervention is associated with a longer duration of EBF (slower cessation).

#### 4.1.2. Interpretation of Key Findings

##### Random Effect

The multilevel survival analysis model significantly predicted exclusive breastfeeding between the intervention (mHealth) and control (received un-intervened care). The random effect showed a moderate variability of 0.10 (95% CI: 0.01–0.07). The likelihood of stopping EBF (/In_p = 0.612082; p = e^/In_p^ = e^0.617373^ ≈ 1.85) increases by 85% as time progresses. The early months of exclusive breastfeeding are associated with a lower risk of stopping (Table 2).

##### Fixed Effects

Compared to the control groups, mothers in the intervention group had a 59% reduced hazard of stopping EBF (AHR = 0.41, 95% CI: 0.26–0.63, *p* < 0.001). Additionally, as ANC frequency increases, the hazard of stopping EBF decreases (AHR = 0.89, 95% CI: 0.80–0.99, *p* < 0.05). Diarrheal disease is associated with a higher hazard of stopping EBF (AHR = 1.40, 95% CI: 1.03–1.90, *p* < 0.05). Women with partners educated to college level or higher had a higher hazard of ending exclusive breastfeeding (AHR = 2.41, 95% CI: 1.01–5.78, *p* < 0.05). Additionally, a family size of 6 to 7 members was associated with a decreased EBF (AHR = 0.62, 95% CI: 0.41–0.93, *p* < 0.05) (Table 2).

### 4.2. Secondary Outcomes

The intervention group had approximately 5 times higher odds of breastfeeding in the first hour after birth (AOR = 4.71, 95% CI: 3.10–7.16, *p* < 0.001). As ANC frequency increases, the likelihood of breastfeeding increases in the first hour after birth (AOR = 1.23, 95% CI: 3.10–7.16, *p* < 0.001). Complications during the pregnancy and delivery were associated with decreased breastfeeding in the first hour after birth (AOR = 0.32, 95% CI: 0.10–0.98, *p* < 0.05) (Table 3).

### 4.3. Model Evaluation

Table 4 illustrates that the final model (Model 3) is the most effective, with a considerable reduction in variability. The comprehensive information about the models was provided in the statistical analysis section (Table 4).

## 5. Discussion

This study assessed the effect of mHealth on EBF in Gamo and Gofa zones in South Ethiopia. The study applied multilevel survival analysis, which provides valuable insights into utilizing mHealth to enhance EBF or other child-feeding practices and factors influencing the duration of exclusive breastfeeding (EBF). EBF in six months and breastfeeding in the first hour after birth in the first month improved. These findings align with global breastfeeding and breastfeeding in the first hour after birth recommendations, highlighting key areas for targeted interventions [25,26,27].

Mothers in the intervention group were less likely to stop EBF before six months compared to the control group (AOR = 0.41). This indicates that the intervention can promote adherence to the WHO recommendation for six months of EBF. The finding is consistent with other studies both in Africa and Ethiopia [17,25,26,27,28]. It underscores the importance of structured interventions, such as breastfeeding education and counseling, in improving EBF rates. Scaling such interventions at a community level could yield widespread benefits for maternal and child health.

Antenatal care (ANC) emerged as an important predictor in both models. Mothers who attended ANC had an 11% lower hazard of stopping exclusive breastfeeding (AHR = 0.89, *p* < 0.05) and were more likely to initiate breastfeeding within the first hour after birth (AOR = 1.23, *p* < 0.001). Other studies suggest that support provided during antenatal care influences breastfeeding [29,30]. UNICEF also recommends that an improved infant and young child feeding practice begin in the first hour of life [31]. ANC visits offer an opportunity for critical education and counseling on infant feeding practices, and emphasis may need to shift toward reinforcing the importance of exclusive breastfeeding for the recommended six months. Strengthening ANC messaging to align with WHO breastfeeding guidelines could further optimize feeding practices.

Mothers who experienced pregnancy complications had a 68% lower likelihood of initiating breastfeeding in the early first hour of life (AOR = 0.32, *p* < 0.05), potentially due to delayed recovery or additional health concerns. Pregnancy complications, particularly during birth, decrease the likelihood of early initiation of breastfeeding in the first hour after birth [32]. Evidence shows that the initiation of breastfeeding after the first hour increases the likelihood of infant mortality [33]. This highlights the importance of focusing on care for complicated births regarding breastfeeding initiation, in addition to information provision.

On the infant side, diarrhea was associated with a 46% higher hazard of stopping exclusive breastfeeding (AHR = 1.47, *p* < 0.05), suggesting that illness episodes may lead to early supplementation with other fluids or foods. Other studies revealed that early complementary feeding is associated with a high risk of diarrheal disease [34,35]. Strengthening community-based counseling on managing infant illness while maintaining breastfeeding could help reduce this risk. Women with a family size of 6–7 members showed a lower hazard of stopping EBF (AHR = 0.62, *p* < 0.05). This suggests that beyond the average family size in Ethiopia (5 ± 2), women tend to practice the recommended feeding practices. Exclusive breastfeeding (EBF) is known to influence fertility regulation, especially by extending postpartum amenorrhea, which can lower the chance of subsequent pregnancies and affect family size [36]. The study suggests that larger family sizes (6–7) facilitate EBF. This might mean that the hazard of EBF rises with family size to a point, but then declines. Mothers may receive more support from family members of these sizes. Evidence from diverse studies suggests that peer support positively promotes exclusive breastfeeding, highlighting the importance of social support networks in sustaining EBF [37,38]. The likelihood of stopping EBF increased among women whose partners had a college degree or higher. The impact of a partner’s or husband’s education on exclusive breastfeeding is complex and varies depending on socioeconomic, cultural, and healthcare access factors. Child feeding practices are a family matter, and women need support from their partners. A positive or negative partner attitude can influence child feeding practices in Ethiopian culture. Higher education levels in men are often linked with both positive and negative maternal and child health outcomes. Wealthier and more educated fathers may encourage formula feeding for convenience, believing it supports the baby’s growth [39,40].

### Limitations and Strengths

The multilevel survival analysis accounts for clustering and identifies both individual- and cluster-level determinants of EBF duration, providing policy-relevant insights for targeted interventions. Some between-cluster variability remains unexplained and could be reduced by incorporating additional contextual factors. Potential issues include the proportional hazards assumption, which may affect robustness, and biases from the measurement and classification of breastfeeding practices. Generalizability is limited, and there may be slight variability in implementation across clusters that warrants caution. Although Ethiopia has widespread mobile subscription coverage (over 70% of the population), restricting the analysis to literate women could limit generalizability. Acknowledge any deviations from the plan and their potential impact, but argue that the deviation is minor and does not undermine conclusions.

## 6. Conclusions

The study reveals that mHealth interventions impact antenatal care, maternal health factors, exclusive breastfeeding (EBF), and early breastfeeding in the first hour of life. Digital health supports breastfeeding and promotes early breastfeeding, suggesting the need for scaling up to larger areas. Antenatal care (ANC) plays a crucial role in shaping feeding behaviors, and maternal health factors such as pregnancy complications and service utilization influence breastfeeding. Sociodemographic factors also influence cultural norms and access to health services.

## 7. Implications

The findings underscore the potential of mHealth interventions to promote breastfeeding while also highlighting gaps in exclusive breastfeeding support. The higher likelihood of initiating breastfeeding within the first hour of life in the intervention group indicates that digital health strategies can be effectively designed to reinforce the WHO recommendation of exclusive breastfeeding for six months before introducing complementary foods.

To optimize breastfeeding promotion, interventions should integrate targeted counseling during antenatal and postnatal care, particularly for mothers with pregnancy complications or infants prone to illness.

Further research is needed to explore the reasons behind the decreased hazard of stopping EBF among mothers with larger family sizes and the role of sociodemographic factors in feeding practices. Strengthening policies and programs that support breastfeeding mothers through health services, workplace policies, and community engagement can further improve infant nutrition outcomes.

## Figures and Tables

**Table 1:** Descriptions of study participants with basic variables.

Variables		Intervention	Controls
		Number (%)	Number (%)
Mode of delivery	Spontaneous vaginal delivery	279(92)	298(95)
	Cesarean Section	23(8)	17(5)
Healthcare provider	Nurse	40(12)	70(21)
	Mid-wife	96(29)	136(40)
	HO	55(16)	66(20)
	Doctor	18(5)	28(8)
	HEW	128(38)	38(11)
Presence of diarrhea	No	286(85)	267(79)
	Yes	51(15)	71(21)
Exclusive breastfeeding	No	91(27)	165(49)
	Yes	246(73)	173(51)

HEW: Health Extension workers; HO: Health Officers.

**Table 2:** Multilevel survival analysis of the effect of mHealth on EBF and associated factors.

Variables	Categories	Adjusted HR/AHR (95%CI)
Treatment group	Controls	1
	Intervention	0.40 (0.26–0.62) ***
Antenatal care		0.87(0.79–0.97) *
Diarrhea	No	1
	Yes	1.47(1.07–2.02) *
Family size	2–3	1
	4–5	0.85(0.61–1.18)
	6–7	0.62(0.41–0.93) *
	8+	0.77(0.42–1.42)
Partner education	Read/write	1
	Primary school (1–8 grade)	1.83(0.78–4.31)
	Secondary school (9–12 grade)	2.15(1.00–5.07)
	College and above	2.41(1.01–5.78) *
_cons		0.13(0.05–0.38) ***
/In_p		0.62(0.50–0.72) ***
Var(_cons)		0.10(0.01–0.07)
LL = −542 Wald chii2(17) = 52, *p* = 0.0002		

Key: * = *p* < 0.05, & *** = *p* < 0.001; EBF: Exclusive Breastfeeding.

**Table 3:** Multilevel analysis of breastfeeding in the first hour after birth.

Variables		Model 0	Model 1	Model 2	Model 3
Antenatal care		-	-	1.25(1.10–1.43) ***	1.23(3.10–7.16) ***
Any complication	No	-	-	1	1
	Yes	-	-	0.32(0.10–1.00) *	0.32(0.10–0.98) *
Marriage	Married and live together	-		1	
	Married and live separately	-		1.07(0.42–2.70)	
Ethnicity	Gamo/Gofa			1	
	Kore			0.68(0.34–1.26)	
	Walaita			1.07(0.42–2.69)	
	Amhara			2.39(0.73–7.76)	
Residences	Rural	-	1	-	
	Urban	-	1.20 (0.77–1.77)	-	
Treatment	Control	-	1	-	1
	Intervention	-	5.20(3.41–7.87) ***	-	4.71(3.10–7.16) ***

Key: * = *p* < 0.05, & *** = *p* < 0.001.

**Table 4:** Evaluation of the subsequent multilevel models.

Random Effect Model Comparison	Model 0	Model 1	Model 2	Model 3
Group-level Variance	0.85	0.22	0.75	0.00
Inter-cluster correlation (ICC)	0.20	0.01	0.18	0.00
Log likelihood ratio (LLR)	−375	−359	−364	−350
AIC BIC	754 763	727 745	746 787	721 766

## Data Availability

The original contributions presented in this study are included in the article. Further inquiries can be directed to the corresponding author.

## Abbreviation

EBF: Exclusive Breastfeeding; BF: Breastfeeding; HFs: Health Facilities; UNICEF: United Nations Children’s Emergency Fund; ANC: Antenatal Care; PNC: Postnatal Care; ICC: Intra-Cluster Correlation; HEWs: Health Extension Workers; AIC: Akaike Information Criterion; BIC: Bayesian Information Criterion; GA: Gestational Age; Wks: Weeks; ODK: Open Data Kit; AOR: and Adjusted Odds Ratio.
